# Anti-IL-20 monoclonal antibody promotes bone fracture healing through regulating IL-20-mediated osteoblastogenesis

**DOI:** 10.1038/srep24339

**Published:** 2016-04-14

**Authors:** Yu-Hsiang Hsu, Yi-Shu Chiu, Wei-Yu Chen, Kuo-Yuan Huang, I-Ming Jou, Po-Tin Wu, Chih-Hsing Wu, Ming-Shi Chang

**Affiliations:** 1 Institute of Clinical Medicine, College of Medicine, National Cheng Kung University, Tainan, Taiwan; 2 Clinical Medicine Research Center, National Cheng Kung University Hospital, Tainan, Taiwan; 3 Department of Biochemistry and Molecular Biology, College of Medicine, National Cheng Kung University, Tainan, Taiwan; 4 Department of Orthopedics, College of Medicine, National Cheng Kung University, Tainan, Taiwan; 5 Department of Family Medicine, College of Medicine, National Cheng Kung University, Tainan, Taiwan

## Abstract

Bone loss and skeletal fragility in bone fracture are caused by an imbalance in bone remodeling. The current challenge in bone fracture healing is to promote osteoblastogenesis and bone formation. We aimed to explore the role of IL-20 in osteoblastogenesis, osteoblast differentiation and bone fracture. Serum IL-20 was significantly correlated with serum sclerostin in patients with bone fracture. In a mouse model, anti-IL-20 monoclonal antibody (mAb) 7E increased bone formation during fracture healing. *In vitro*, IL-20 inhibited osteoblastogenesis by upregulating sclerostin, and downregulating osterix (OSX), RUNX2, and osteoprotegerin (OPG). IL-20R1 deficiency attenuated IL-20-mediated inhibition of osteoblast differentiation and maturation and reduced the healing time after a bone fracture. We conclude that IL-20 affects bone formation and downregulates osteoblastogenesis by modulating sclerostin, OSX, RUNX2, and OPG on osteoblasts. Our results demonstrated that IL-20 is involved in osteoregulation and anti-IL-20 mAb is a potential therapeutic for treating bone fracture or metabolic bone diseases.

IL-20 is a member of the IL-10 family, which includes IL-10, -19, -20, -22, -24, and -26^1^,^2^. These members share 18–25% amino acid identity with IL-10^3^. IL-20 is expressed in monocytes, epithelial cells, and endothelial cells. It acts on multiple cell types by activating a heterodimer receptor complex of either IL-20R1/IL-20R2 or IL-22R1/IL-20R2^4^. It is also involved in various inflammatory diseases, such as psoriasis^1^,^5^,^6^, rheumatoid arthritis^7^,^8^, and osteoporosis^9^.

Bone fracture healing is closely linked with both formation and remodelling. Endochondral bone formation usually occurs in fracture healing^10^. Undifferentiated mesenchymal cells secrete a cartilage like matrix which is mineralized. The matrix is invaded by vascular buds which bring osteoclasts and osteoblasts to the area. The osteoclasts absorb the calcified matrix and it is the osteoblasts that lay down new bone. In essence, a cartilage matrix is replaced by bone^11^. Primary bone formation is followed by remodeling, in which the initial bony callus is reshaped by secondary bone formation and resorption to restore the anatomical structure that supports mechanical loads^10^,^12^. Two major bone cells are involved in bone remodeling: bone forming cells (osteoblasts) and bone resorbing cells (osteoclasts)^13^,^14^,^15^. The major events that trigger osteoblastogenesis and osteoclastogenesis are the transition of mesenchymal stem cells (MSC) into osteoblasts, and the transition of monocyte/macrophage precursors into osteoclasts^16^. Dysregulation of osteoblast and osteoblast differentiation is involved in the pathogenesis of skeletal diseases like osteoporosis and bone fracture^17^,^18^,^19^,^20^. Bone-forming osteoblasts are derived from MSC^21^. Runt-related transcription factor 2 (RUNX2), osterix (OSX), and β-catenin are essential factors for osteoblast differentiation to activate specific signaling pathways in MSC and osteoprogenitor cells^21^,^22^.

Sclerostin encoded by the *SOST* gene, is a secreted glycoprotein that negatively regulates bone formation. Sclerostin inhibits osteoblast differentiation and mineralization *in vitro*, and mice that overexpress sclerostin exhibit an osteoporotic phenotype^23^,^24^,^25^. Sclerostin inhibits Wnt/β-catenin signaling and reduces bone formation by inhibiting osteoblast differentiation, proliferation, and function^26^. Sclerostin knockout mice have a phenotype with a high bone mass, as do humans who have sclerosteosis and Van Buchem disease^27^. Preclinical data showed that anti-sclerostin antibody reversed estrogen-deficiency-induced bone loss by increasing bone formation and bone mass in an ovariectomized (OVX) rat model^28^.

We previously^7^,^8^,^9^ showed that IL-20 and its receptors are all expressed on osteoclasts, osteoblasts, and rheumatoid synovial fibroblasts. IL-20 promoted osteoclast differentiation and blockading IL-20 might provide a novel therapeutic approach for rheumatoid arthritis and osteoporosis, which support the notion that IL-20 is important for regulating bone homeostasis. We hypothesize that IL-20 is involved in osteoblastogenesis and in the maturation of osteoblasts. Therefore, we explored whether IL-20 regulates osteoblast differentiation and assessed the effects of IL-20 blockade in bone fracture mouse model.

## Results

### Anti-IL-20 monoclonal antibody increased bone formation during fracture healing *in vivo*

**Figure 1:**
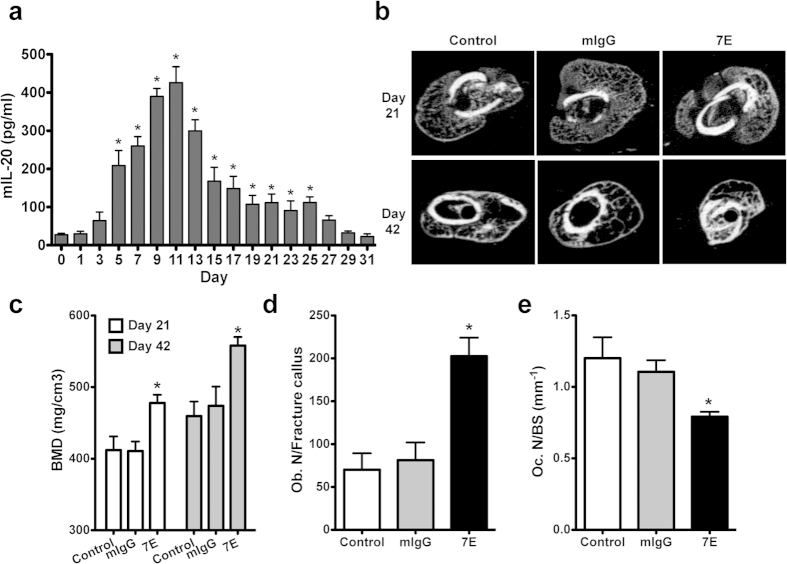
IL-20 was involved in osteoblastogenesis during bone fracture healing *in vivo.* (**a**) Levels of IL-20 in serum from mice with bone fracture were analyzed at the indicated time during fracture healing. Values are means ± SD of 6 mice. Data are representative of three independent experiments. *P < 0.05 versus 0 day post-fracture. (**b**) Representative figures of micro-CT analyses of the fracture callus of mice 21 and 42 days post-fracture, followed by no treatment (Control), 3 mg mIgG/kg/3 d treatment, and 3 mg 7E/kg/3 d treatment (*n* = 8/group). Images of these individual slices were taken from the central region of each callus. Data are representative of three independent experiments. (**c**) BMD at the fracture callus was assessed using micro-CT scans 21 and 42 days post-fracture (*n* = 8/group). Values are means ± SD. Data are representative of three independent experiments. *P < 0.05 versus mIgG controls. (**d**) ALP staining analysis and quantification of the number of osteoblasts in fracture callus 21 days post-fracture (*n* = 8/group). Values are means ± SD of 3 frozen sections. Data are representative of three independent experiments. *P < 0.05 versus mIgG controls. (**e**) TRAP staining analysis and quantification of the number of osteoclasts in bone surface 21 days post-fracture (*n* = 8/group). Values are means ± SD of 3 frozen sections. Data are representative of three independent experiments. *P < 0.05 versus mIgG controls.

Our previous finding demonstrated that IL-20 blockade by anti-IL-20 monoclonal antibody (anti-IL-20 mAb, 7E) inhibited osteoclast differentiation^9^. The effect of IL-20 in osteoblast-mediated bone formation, however, was not well understood. We hypothesized that IL-20 could mediate bone formation through regulating the balance of osteoblastogenesis and osteoclastogenesis. To clarify the *in vivo* role of IL-20 in osteoblast differentiation, we generated a mouse model of bone fracture to analyze whether IL-20 is associated with bone fractures. IL-20 was locally expressed on osteoclasts, osteoblasts, and chondrocyte of the fracture callus (data not shown). ELISA showed that IL-20 serum level was significantly upregulated in mice with bone fractures between 5 and 25 days post-fracture (Fig. 1a), indicating that IL-20 might be involved in bone fracture healing. In mice treated with mIgG and 7E, respectively, the fracture areas in radiographs were not significantly different 10–42 days post-fracture (Supplementary Fig. 1). However, micro-CT scans of the mouse fracture callus showed a higher BMD in 7E-treated mice than in mIgG-treated mice between 21 and 42 days post-fracture (21day: 477.83 ± 11.42 versus 410 ± 13.39, 42 day: 558.5 ± 12.46 versus 473.83 ± 26.81, Fig. 1b,c). Bone histomorphometric analyses at 21 days post-fracture demonstrated that compared with mIgG-treated mice, 7E-treated mice significantly increased the ratios of bone volume/tissue volume (BV/TV), trabecular bone thickness (Tb.Th), and trabecular number (Tb.N.) in the fracture site (Supplementary Fig. 2). The values of bone formation-related parameters (mineral apposition rate; MAR and bone formation rate; BFR) were increased in 7E-treated mice compared to mIgG-treated mice (Supplementary Fig. 2). More osteoblasts were observed in the fracture callus of 7E-treated mice compared to mIgG-treated mice (Fig. 1d and Supplementary Fig. 3). TRAP staining showed that lower osteoclast number in the bone surface of 7E-treated mice compared to mIgG-treated mice (Fig. 1e), which suggested that IL-20 was detrimental in fracture healing and that blocking of IL-20 by 7E promoted bone formation during fracture healing via increasing osteoblastogensis and decreasing osteoclastogenesis.

### IL-20R1 deficiency enhanced osteoblast differentiation and reduced the healing time after a bone fracture

**Figure 2:**
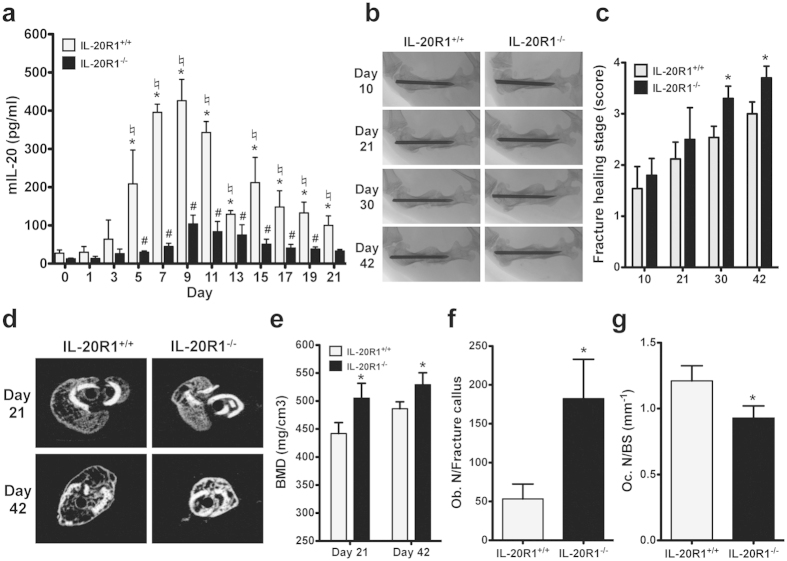
IL-20R1 deficiency increased osteoblast differentiation and promoted fracture healing by increasing BMD in the mouse fracture model. (**a**) Levels of IL-20 in serum from IL-20R1^+/+^ and IL-20R1^−/−^ mice with bone fracture were analyzed at the indicated times during fracture healing. Values are means ± SD of 6 mice. Data are representative of three independent experiments. *P < 0.05 versus IL-20R1^+/+^ mice 0 day post-fracture. ^#^*P* < 0.05 versus IL-20R1^−/−^ mice 0 day post-fracture.^♮^*P* < 0.05 versus IL-20R1^−/−^ mice post-fracture. (**b**) Radiographs of fractured right femurs from IL-20R1^+/+^ and IL-20R1^−/−^ mice (*n* = 8/group). The radiographic time course is shown at the callus formation for the mice in each group. Data are representative of three independent experiments. (**c**) Radiological assessments on fracture healing by fracture healing stage score of the X-ray images from IL-20R1^+/+^ and IL-20R1^−/−^ mice (*n* = 8/group). Data are representative of three independent experiments. (**d**) Representative figures of micro-CT analyses of the fracture callus of IL-20R1^+/+^ and IL-20R1^−/−^ mice 21 and 42 days post-fracture (*n* = 8/group). Images of these individual slices were taken from the central region of each callus. Data are representative of three independent experiments. (**e**) BMD at the fracture line was assessed using micro-CT scans 21 and 42 days post-fracture (*n* = 8/group). Values are means ± SD. Data are representative of three independent experiments. *P < 0.05 versus IL-20R1^+/+^ mice. (**f**) ALP staining analysis and quantification of the number of osteoblasts in the fracture callus 21 days post-fracture (*n* = 8/group). Values are means ± SD of 3 frozen sections. Data are representative of three independent experiments. *P < 0.05 versus IL-20R1^+/+^ mice. (**g**) TRAP staining analysis and quantification of the number of osteoclasts in bone surface 21 days post-fracture (*n* = 8/group). Values are means ± SD of 3 frozen sections. Data are representative of three independent experiments. *P < 0.05 versus IL-20R1^+/+^ mice.

We also created a bone fracture model in IL-20R1^−/−^ mice to investigate the role of IL-20R1 signaling in fracture healing. Although serum IL-20 level was upregulated in both IL-20R1^+/+^ and IL-20R1^−/−^ mice after bone fracture, we found that IL-20 level was significantly higher in IL-20R1^+/+^ mice than in IL-20R1^−/−^ mice between 5 and 21 days post-fracture (Fig. 2a). X-rays showed that, 21 days post-fracture, IL-20R1^−/−^ mice had a denser callus that persisted for the remainder of the experiment (Fig. 2b). Fractures healed significantly more quickly in IL-20R1^−/−^ mice than in IL-20R1^+/+^ mice (Fig. 2c). IL-20R1^−/−^ mice had significantly higher BMD and greater bone formation than did IL-20R1^+/+^ mice at 21 days (506.22 ± 12.37 versus 441.78 ± 19.59) and 42 days (521 ± 21.48 versus 485.11 ± 26.51) post-fracture (Fig. 2d,e and Supplementary Fig. 4). Furthermore, IL-20R1^−/−^ mice had more osteoblasts in fracture callus than did IL-20R1^+/+^ mice at 21 days post-fracture (Fig. 2f and Supplementary Fig. 5). TRAP staining also showed lower osteoclast number in bone surface of IL-20R1^−/−^ mice compared to IL-20R1^+/+^ mice (Fig. 2g). These findings demonstrated that IL-20R1 signaling was inhibitory for osteoblastogenesis and IL-20R1 deficiency enhanced osteoblast differentiation and reduced the healing time after a bone fracture.

### IL-20 was significantly correlated with serum sclerostin in patients with bone fracture and osteoporosis

**Figure 3:**
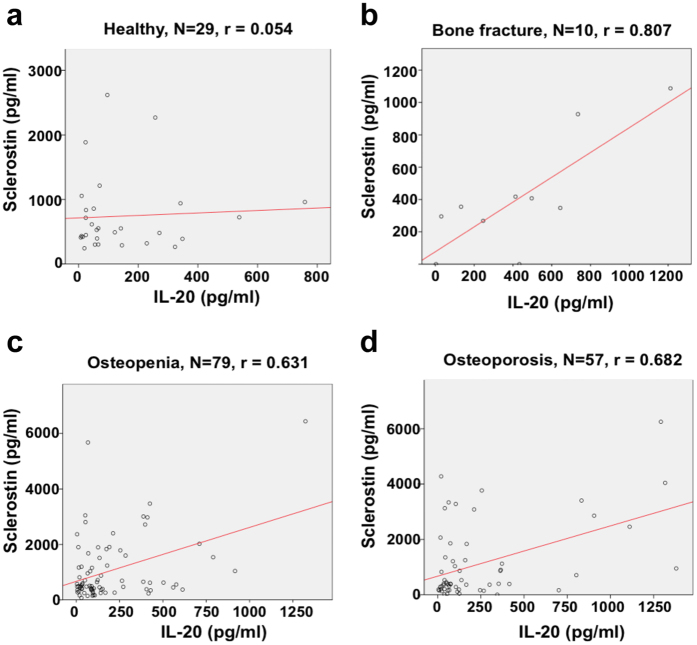
Serum IL-20 level was associated with serum sclerostin level in patients with bone fracture, osteopenia, and osteoporosis. Level of IL-20 and sclerostin in serum from (**a**) 29 healthy volunteers, (**b**) 10 patients with bone fracture, (**c**) 79 patients with osteopenia, and (**d**) 57 patients with osteoporosis was analyzed. Values ≥0 and ≤0.3: weak positive linear relationship via a shaky linear rule; from >0.3 and ≤0.6: moderate positive linear relationship via a fuzzy-firm linear rule; >0.6 and ≤1.0: strong positive linear relationship via a firm linear rule.

Our results demonstrated that IL-20 signaling was inhibitory for osteoblastogenesis *in vivo* (Figs 1 and 2). Sclerostin inhibits osteoblastogenesis that highly correlates with bone loss-related diseases^23^,^24^,^25^. To confirm the clinical correlation between IL-20 and sclerostin in patients with bone fracture, we collected their serum and used ELISA to analyze their IL-20 and sclerostin. Linear regression analysis showed that IL-20 was not correlated with sclerostin in healthy volunteers (r = 0.054, Fig. 3a), but positively correlated with sclerostin in patients with bone fracture (r = 0.807, Fig. 3b). The findings were similar in patients with osteopenia (r = 0.631, Fig. 3c) and osteoporosis (r = 0.682, Fig. 3d), which suggested that IL-20 may be involved in osteoblastogenesis by regulating sclerostin, and associated with metabolic bone diseases.

**Figure 4:**
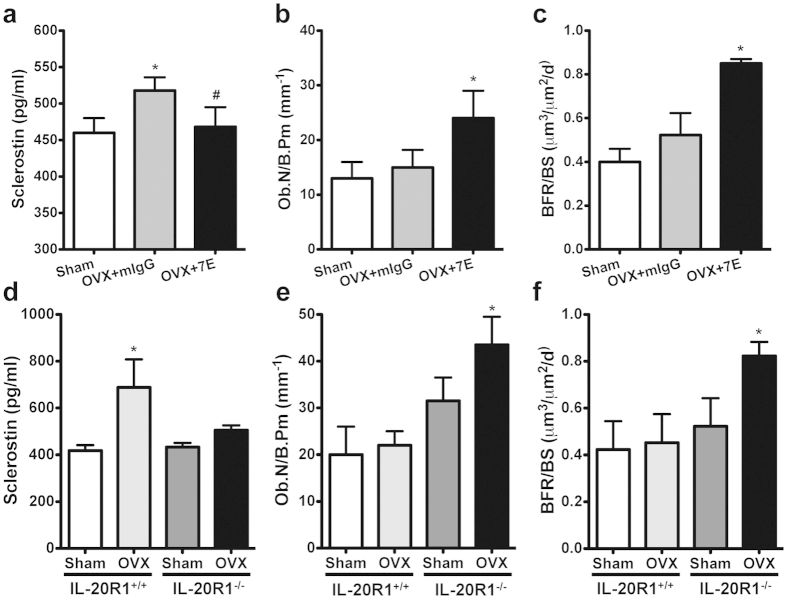
IL-20 signaling regulated osteoblasts by modulating sclerostin in the OVX-induced bone loss model. (**a**) Serum levels of sclerostin in Sham and OVX mice treated with 3 mg mIgG/kg/3 d treatment, or 3 mg 7E/kg/3 d treatment were analyzed (*n* = 5/group). Values are means ± SD. Data are representative of three independent experiments. *P < 0.05 versus Sham controls. ^#^*P* < 0.05 versus mIgG controls. (**b**) ALP staining analysis of mice tibia 8 weeks in Sham and OVX treated with 3 mg mIgG/kg/3 d treatment, or 3 mg 7E/kg/3 d treatment (*n* = 5/group). Quantification of the number of osteoblasts per bone perimeter (Ob.N/B.Pm). Values are means ± SD of 3 frozen sections. Data are representative of three independent experiments. *P < 0.05 versus mIgG controls. (**c**) Analysis of dynamic bone histomorphometric parameters (bone formation rate/bone surface; BFR/BS) in the distal femur collected from the groups of mice indicated. Values are means ± SEM. Data are representative of three independent experiments. *P < 0.05 versus mIgG controls. (**d**) Serum level of sclerostin in IL-20R1^+/+^ and IL-20R1^−/−^ mice was analyzed using an ELISA kit 8 weeks after an OVX or Sham-operated control (*n* = 5/group). Values are means ± SD. Data are representative of three independent experiments. *P < 0.05 versus Sham-IL-20R1^+/+^ mice. (**e**) ALP staining analysis of the tibias of IL-20R1^+/+^ and IL-20R1^−/−^ mice 8 weeks after an OVX or Sham-operated control (*n* = 5/group). Values are means ± SD of 3 frozen sections. *P < 0.05 versus Sham-IL-20R1^−/−^ mice. (**f**) Analysis of dynamic bone histomorphometric parameters (BFR/BS) in the distal femur collected from the groups of mice indicated. Values are means ± SEM. Data are representative of three independent experiments. *P < 0.05 versus IL-20R1^+/+^ mice.

We generated OVX-induced osteoporotic mice to clarify whether 7E could regulate sclerostin in OVX-induced bone loss. ELISA showed that serum sclerostin was upregulated in the OVX (mIgG-treated control) mice but downregulated in 7E-treated OVX mice (516.55 ± 43.49 versus 460.45 ± 32.67, Fig. 4a). We previously reported that lower osteoclast numbers (N.Oc/BS) and smaller osteoclast areas of bone surface (Oc.S/BS) were observed in 7E-treated OVX mice^9^. In the present study, we found that 7E-treated OVX mice had more osteoblasts than did mIgG-treated OVX mice (24.67 ± 9.01 versus 15 ± 4.69, Fig. 4b). The value of bone formation rate was also increased in 7E-treated OVX mice compared to mIgG-treated mice (Fig. 4c). In addition, ELISA confirmed that sclerostin secretion was upregulated in OVX-IL-20R1^+/+^ mice but not in OVX-IL-20R1^−/−^ mice (Fig. 4d). Osteoblast numbers in OVX-IL-20R1^−/−^ mice were significantly higher than in sham-IL-20R1^−/−^ mice (44 ± 3.5 versus 31 ± 4, Fig. 4e). The value of bone formation rate was also increased in OVX-IL-20R1^−/−^ mice compared to sham-IL-20R1^−/−^ mice (Fig. 4f). This finding revealed a pivotal role of IL-20/IL-20R1 signaling in negative regulation of osteoblast differentiation and that deficiency of IL-20/IL-20R1 signaling promoted bone formation during metabolic bone disease.

### 7E promoted osteoblast differentiation

**Figure 5:**
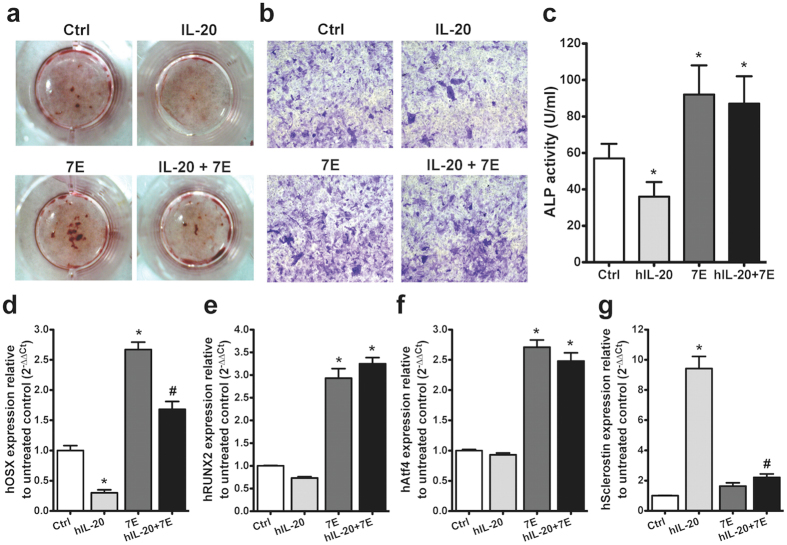
7E promoted osteoblast differentiation in hAFSCs. (**a,b**) hAFSCs were incubated with osteoblast differentiation medium. The osteoblasts were characterized using ALP staining (**a**) 14 days post-incubation, or Alizarin staining (**b**) 28 days post-incubation. (**c**) Cell lysates were collected and measured; ALP activity was measured using an assay kit 14 days post-incubation. Values are means ± SD. *P < 0.05 versus untreated controls (Ctrl). (**d–f**) hAFSCs were cultured under osteogenic conditions for 14 days. mRNA was isolated and the transcripts of OSX, RUNX2, and Atf4 were measured using RTQ-PCR with specific primers. The quantification analysis of mRNA was normalized; GAPDH was the housekeeping gene. *P < 0.05 versus untreated controls (Ctrl). ^#^*P* < 0.05 versus the 7E-treated group. (**g**) hAFSCs were cultured under osteogenic conditions for 28 days. The expression of sclerostin was analyzed using RTQ-PCR with specific primers. The quantification analysis of mRNA was normalized; GAPDH was the housekeeping gene. *P < 0.05 versus untreated controls. ^#^*P* < 0.05 versus the IL-20-treated group. All experiments were run three times, with similar results. Data are from a representative experiment.

The *in vivo* study revealed that IL-20 was associated with bone formation in the model of bone fracture and osteoporosis. Our previous finding indicated that IL-20 is an enhancing factor for osteoclast differentiation^9^. Human amniotic fluid-derived stem cells (hAFSCs) could be induced to differentiate into adipocytes, osteocytes and neuronal cells^29^,^30^,^31^. To further delineate the role of IL-20 in osteoblastogensis, we used hAFSCs to evaluate whether IL-20 regulates the differentiation of osteoblast from pluripotent stem cell. Immunohistochemical staining and reverse transcriptase-polymerase chain reaction (RT-PCR) showed that IL-20 and its receptor subunits (IL-20R1, IL-20R2, and IL-22R1) were expressed in hAFSCs (Supplementary Fig. 6a,b), which indicated that hAFSCs could be the target cells of IL-20 in an autocrine manner. To further examine the effect of IL-20 and 7E on osteoblasts, we cultured hAFSCs with IL-20, 7E, or IL-20 plus 7E under osteogenic differentiation conditions for 14 and 28 days. Less ALP staining was observed in the IL-20-treated group than that in the untreated control cultured for 14 days, while much more ALP staining was observed in 7E-treated group (Fig. 5a). Alizarin red S staining showed that bone nodule formation was downregulated in IL-20-treated hAFSCs, whereas it was upregulated in 7E-treated hAFSCs cultured for 28 days (Fig. 5b), suggesting that IL-20 inhibited osteoblast differentiation. ALP activity was increased in the 7E-treated group than in the control group (91.34 ± 17.69 versus 53.14 ± 10.16, Fig. 5c), which suggested that endogenous IL-20 is crucial for osteoblast differentiation. In addition, Real-time quantitative (RTQ)-PCR showed that 7E markedly upregulated the mRNA expressions of the osteoblast differentiation markers OSX, RUNX2, and activating transcription factor 4 (Atf4) *in vitro* (Fig. 5d–f), which was evidence that endogenous secretion of IL-20 is crucial when hAFSCs undergo osteoblastic lineage progression. Furthermore, hAFSCs were cultured under osteogenic conditions for 28 days to differentiate into mature osteocytes. The expression of sclerostin was upregulated by IL-20 in hAFSC-derived mature osteocytes (Fig. 5g), which suggested that IL-20 could inhibit osteoblastogenesis through downregulating the osteoblast differentiation factors (OSX, RUNX2, and Atf4) and upregulating the anti-osteoblastogenic factor, sclerostin.

### IL-20 is an inhibitory factor for osteoblast maturation

**Figure 6:**
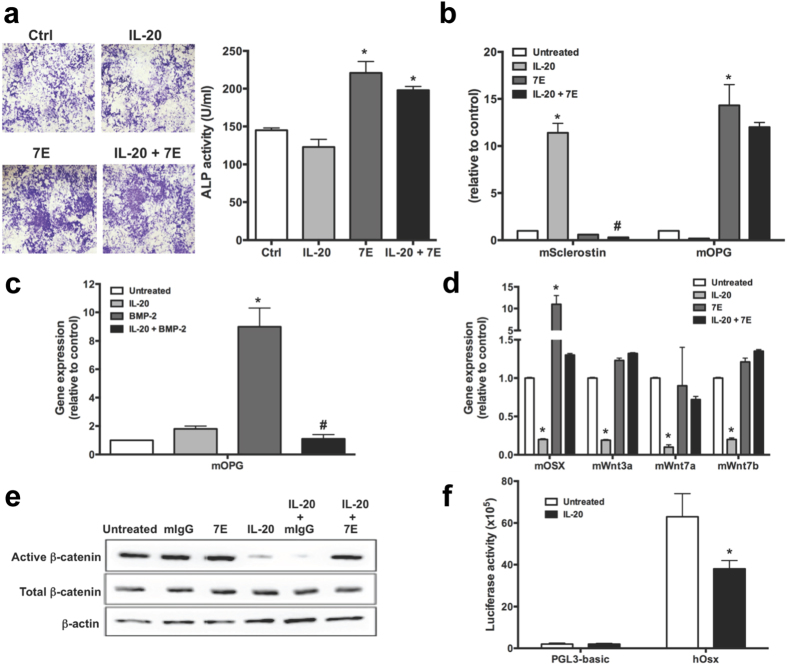
Blocking of IL-20 by anti-IL-20 mAb 7E promoted osteoblast maturation and upregulated osteoblastogenic factors in MC3T3-E1 cells. (**a**) MC3T3-E1 cells were incubated with osteoblast differentiation medium. Their ability to differentiate into osteoblasts was characterized using ALP staining with an assay kit 14 days post-incubation. *P < 0.05 versus untreated controls (Ctrl). (**b**) MC3T3-E1 cells were treated with IL-20 (200 ng/ml), 7E (2 μg/ml), or IL-20+7E for 4 h. Sclerostin and OPG expression were analyzed using RTQ-PCR with specific primers. The quantification analysis of mRNA was normalized; GAPDH was the housekeeping gene. *P < 0.05 versus untreated controls. ^#^*P* < 0.05 versus the IL-20-treated group. (**c**) MC3T3-E1 cells were pre-incubated with BMP-2 for 2 h, and then treated with IL-20 (200 ng/ml) for another 4 h. OPG expression levels were analyzed using RTQ-PCR with specific primers. The quantification analysis of mRNA was normalized; GAPDH was the housekeeping gene. *P < 0.05 versus untreated controls. ^#^*P* < 0.05 versus the BMP-2-treated group. All experiments were run three times, with similar results. Data are representative of 3 independent experiments. (**d**) MC3T3-E1 cells were treated with IL-20 (200 ng/ml), 7E (2 μg/ml), or IL-20+7E for 4 h. mRNA was isolated and the transcripts of OSX, Wnt3a, Wnt7a, and Wnt7b were analyzed using RTQ-PCR with specific primers. The quantification analysis of mRNA was normalized; GAPDH was the housekeeping gene. *P < 0.05 versus untreated controls. All experiments were run three times, with similar results. Data are from a representative experiment. (**e**) MC3T3-E1 cells were incubated with mIgG (2 μg/ml), 7E (2 μg/ml), IL-20 (200 ng/ml), IL-20+mIgG or IL-20+7E for 6 h, and cell lysates were collected and analyzed using Western blotting for the indicated protein. Data are representative of three independent experiments. (**f**) HEK293 cells transfected with human OSX promoter construct and incubated with IL-20 (200 ng/ml) for 24 h. Cell lysate were collected and analyzed using a luciferase assay. *P < 0.05 versus untreated controls. All experiments were run three times, with similar results. Data are from a representative experiment.

To determine whether IL-20 participates in the development and maturation from preosteoblast to mature osteoblast, we used preosteoblastic MC3T3-E1 cells for *in vitro* osteoblast maturation. The cells were cultured with IL-20, 7E, or IL-20 plus 7E under osteogenic conditions for 14 days. ALP staining showed that 7E upregulated, whereas IL-20 slightly decreased the differentiation of MC3T3-E1 cells into osteoblasts. In addition, 7E-treated group also showed a higher ALP activity than that in the untreated control (Ctrl) (217 ± 28.61 versus 144.96 ± 11.00, Fig. 6a). These results indicated that the endogenous expression IL-20 in this culture system acts an inhibitory factor for osteoblast maturation.

### IL-20 targeted osteoblasts and upregulated sclerostin expression

The expression of sclerostin was upregulated in the presence of IL-20 during the differentiation of hAFSCs into osteoblasts (Fig. 5g), we hypothesized that IL-20 may also target osteoblast progenitors and induces sclerostin expression, which inhibits osteoblast differentiation. To confirm this possibility, pre-osteoblastic MC3T3-E1 cells were cultured with IL-20, 7E or IL-20 plus 7E. RTQ-PCR showed that sclerostin expression was significantly higher in IL-20-treated-MC3T3-E1 cells than that in untreated cells, whereas 7E completely inhibits IL-20-induced sclerostin expression when the cells were co-treated with IL-20 and 7E (Fig. 6b). These *in vitro* results demonstrated that IL-20 is an anti-osteoblastogenesis factor and an upstream mediator of sclerostin.

### IL-20 regulated OPG expression in osteoblasts

OPG, a soluble decoy receptor of RANKL, is synthesized by osteoblasts^32^. We further analyzed whether IL-20 regulates OPG expression in MC3T3-E1 cells. RTQ-PCR showed that IL-20 treatment alone did not significantly affect OPG expression compared to the untreated control. Interestingly, 7E upregulated OPG expression in MC3T3-E1 cells, suggesting that endogenous IL-20 is inhibitory for OPG expression (Fig. 6b). We hypothesized that endogenously-expressed basal level of IL-20 is high enough to suppress the OPG level in osteoblasts. To test this possibility, we treated MC3T3-E1 cells for 2 h with BMP-2, an OPG-inducing factor, and then incubated the cells with IL-20 for 4 h. RTQ-PCR showed that BMP-2-induced OPG expression was inhibited by IL-20 (Fig. 6c). This finding confirmed that minimum expression level of endogenous IL-20 is inhibitory for OPG expression in osteoblasts and that IL-20 inhibits differentiation of preosteoblasts into mature osteoblasts via regulating the balance of pro-and anti-osteogenic factors.

### IL-20 regulated transcription factors associated with osteoblastogenesis

We next tested whether IL-20 inhibited osteoblastogenesis-related factors. MC3T3-E1 cells were treated with IL-20 for 4 h. RTQ-PCR showed that OSX, Wnt3a, Wnt7a, and Wnt7b transcripts (Fig. 6d) were significantly lower in IL-20-treated cells, which indicated that IL-20 regulated OSX through the canonical Wnt/β-catenin pathway. To confirm that IL-20 regulated osteoblastogenesis through the Wnt/β-catenin pathway, we treated MC3T3-E1 cells with IL-20 or 7E and analyzed the active β-catenin protein level using Western blotting. The production of active β-catenin in MC3T3-E1 cells was inhibited after treatment with IL-20 (Fig. 6e).

### IL-20 suppressed OSX promoter activity

To demonstrate whether transcription factor was regulated by IL-20 during osteoblastogenesis, analysis of the human RUNX2 P1, RUNX2 P2 and OSX promoter activity was performed using luciferase reporter constructs in a HEK293 cells. IL-20 treatment in HEK293 cells downregulated the promoter activity of OSX (Fig. 6f), while the activity of RUNX2 P1 and RUNX2 P2 were not affected (data not shown). These data suggest IL-20 inhibited osteoblast differentiation through downregulating the OSX promoter activity.

### IL-20R1 deficiency enhanced osteoblast differentiation and maturation

**Figure 7:**
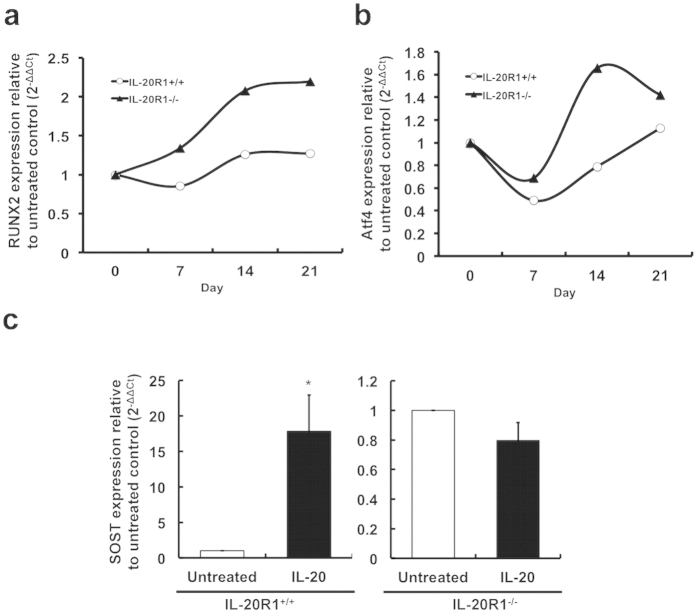
IL-20R1 deficiency enhanced osteoblastogenic gene expression. Primary mouse preosteoblastic calvaria cells were isolated from 24-h-old IL-20R1^+/+^ and IL-20R1^−/−^ mice and cultured under osteogenic conditions for 21 days. mRNA was isolated, and (**a**) RUNX2 and (**b**) Atf4 transcripts were analyzed using RTQ-PCR with specific primers. All experiments were run three times, with similar results. Data are from a representative experiment. (**c**) Mature osteoblasts derived from IL-20R1^+/+^ and IL-20R1^−/−^ mice were incubated with IL-20 (200 ng/ml) for 6 h. mRNA was isolated and sclerostin transcripts were analyzed using RTQ-PCR with specific primers. *P < 0.05 versus IL-20R1^+/+^ untreated cells. All experiments were run three times, with similar results. Data are from a representative experiment.

To confirm IL-20’s function in osteoblast differentiation, we isolated and cultured preosteoblastic calvaria cells from newborn IL-20R1^+/+^ and IL-20R1^−/−^ mice under osteogenic conditions for 21 days. Osteoblast differentiation markers such as RUNX2 and Atf4 were markedly upregulated in IL-20R1^−/−^ cells (Fig. 7a,b). Moreover, IL-20R1^+/+^ osteoblasts produced more sclerostin in response to IL-20 than did IL-20R1^−/−^ osteoblasts (Fig. 7c). These results indicated that IL-20R1 deficiency enhanced osteoblast differentiation and maturation through upregulating the osteogenic genes RUNX2, Atf4 and inhibiting the anti-osteogenic gene sclerostin.

## Discussion

**Figure 8:**
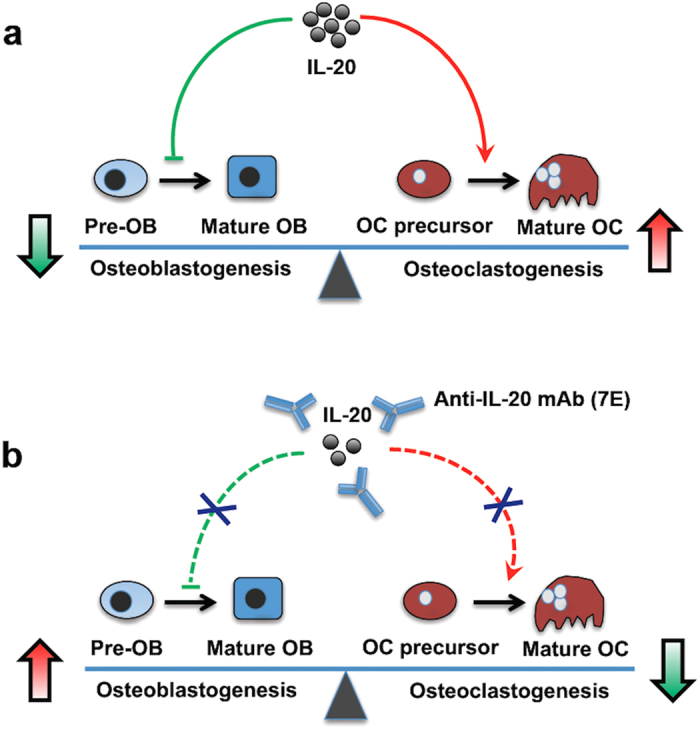
Working model for the activities of IL-20 and 7E in osteogenesis. (**a**) A schematic representation showing that IL-20 is an inhibitory factor for osteoblastogenesis and promotes osteoclastogenesis. (**b**) Blocking of IL-20 by anti-IL-20 mAb (7E) caused the increased osteoblastogenesis and the inhibited osteoclastogenesis.

The present study provides new evidence that IL-20 inhibits osteoblastogenesis by regulating sclerostin, OSX, RUNX2, and OPG on osteoblasts. We identified a pivotal role of IL-20 in osteoblast differentiation. Anti-IL-20 mAb (7E) not only downregulates osteoclast formation^9^ but also upregulates osteoblast formation. Furthermore, IL-20-mediated inhibition in osteoblast differentiation and maturation *in vitro* and *in vivo* were inhibited in IL-20R1^−/−^ mice. We therefore conclude that IL-20R1 is important in IL-20-mediated osteoblastogenesis, and that IL-20 is pivotal in maintaining the balance of osteoclast differentiation and osteoblast differentiation. We hypothesized a working model for the activities of IL-20 and 7E: IL-20 is a negative regulator in bone homeostasis and that the 7E, which inhibits osteoclastogenesis and promotes osteoblastogenesis. Therefore, 7E enhances BMD, serves the therapeutic potential for treating osteoporosis, bone fracture and other metabolic bone diseases (Fig. 8a,b).

Bone formation is characterized by a sequence of events starting with the commitment of osteoprogenitor cells and their differentiation into preosteoblasts and then into mature osteoblasts whose function is to synthesize the bone matrix that becomes progressively mineralized. hAFSC can be differentiated into an osteoblastic lineage under osteogenic conditions. We found that IL-20 and its receptors were expressed in hAFSC. IL-20, in an autocrine manner, targeted stem cells and inhibited the expression of OSX. In addition, IL-20 inactivated osteoblastogenic signaling and 7E promoted the activation of OSX and RUNX2, the master regulators for osteoblast differentiation. Therefore, IL-20 contributes to osteoblastogenesis via OSX-, and RUNX2-induced activity. 7E increased osteoblast differentiation, which supports our hypothesis. IL-20 upregulated sclerostin expression in hAFSC-derived mature osteoblasts and osteocytes. This suggested that IL-20 not only acted on the stem cells to regulate the initiation of osteoblast differentiation, but also targeted mature osteoblasts to inhibit osteoblastogenesis by regulating sclerostin.

The RANKL-RANK signaling mechanism is one of the major pathways of osteoclast formation and activity. The other essential regulating component of the RANK/RANKL system is OPG. OPG is a soluble decoy receptor of RANKL and is synthesized by osteoblasts. Although OPG expression was not significantly different between the IL-20-treated and untreated MC3T3-E1 cells in the present study, 7E highly upregulated OPG expression in MC3T3-E1 cells, which indicated that the minimal level of IL-20 is enough to suppress OPG expression. Therefore, we hypothesized that IL-20 is endogenously expressed in small amount to suppress the OPG level in osteoblasts. That IL-20 inhibited BMP-2-induced OPG expression in MC3T3-E1 cells confirmed our hypothesis. Our previous study also demonstrated that IL-20 upregulated RANKL in MC3T3-E1 cells and primary mature osteoblasts^9^. Therefore, we conclude that IL-20 modulates the expression of RANKL and OPG in osteoblasts, increases the ratio of these two factors, then controls osteoclast differentiation and, consequently, bone remodeling. Even though MC3T3-E1 cells did not express significant levels of sclerostin, which is an osteocyte marker in late stage and involved in the regulation of bone formation, sclerostin was shown to be induced in MC3T3-E1 cells by the addition of osteogenic growth factors^33^,^34^. Our results showed that IL-20 also induced sclerostin expression in MC3T3-E1 cells. Therefore, sclerostin is inducible in osteoblasts under specific condition.

Fracture repair is a continuum of morphological phases that can be measured by multiple approaches to assess the progress of healing^35^. Changes in callus composition and cellular activity with either genetic manipulation or therapeutic intervention may reflect an important role for the target protein in fracture repair^36^. In this study, radiography showed that the callus was denser in IL-20R1^−/−^ mice at day 21, an effect that persisted through day 42. These changes were most likely due to increase bony tissues within the callus of IL-20R1^−/−^ mice. In mouse fracture model, we observed serum IL-20 level was upregulated in both IL-20R1^+/+^ and IL-20R1^−/−^ mice. This may be attributed to the alternative signaling through IL-22R1/IL-20R2 receptor complex. Expression of all the three receptor subunits on osteoblasts suggested this possibility. Moreover, we found that IL-20 level was significantly higher in IL-20R1^+/+^ mice than in IL-20R1^−/−^ after bone fracture, which indicated that IL-20 is autoregulated by itself through IL-20R1/IL-20R2 receptor complex signaling and secretes more IL-20 in the bone microenvironment during fracture healing process. IL-20R1^−/−^ mice had a shorter time for fracture healing confirmed our hypothesis that IL-20 targets osteoblast and slows down the fracture healing through IL-20R1 signaling. In addition, IL-20R1^−/−^ mice had significantly higher BMD and greater bone formation. These results further supported that IL-20 played important role in osteoblast differentiation and that IL-20/IL-20R1 signaling was critical for regulating BMD and bone formation during metabolic bone disease.

Choices of established palliative and disease-modifying therapies are available for osteoporosis. None of them are curative, however; they are only partially effective for slowing down or stopping disease progression. Pharmacological agents for treating osteoporosis may be classified as antiresorptive or osteoanabolic, depending on whether the principal means of improving bone strength is by inhibiting osteoclastic bone resorption or stimulating osteoblastic bone formation. RANKL inhibitors such as OPG and anti-RANKL antibody are inhibitors of bone resorption, presumably because of their effects on osteoclasts. One therapeutic drug used to treat osteoporosis is denosumab, an anti-RANKL mAb^37^,^38^. The sclerostin inhibitor AMG 785 (anti-sclerostin mAb) stimulated bone formation and improved the strength of the fracture callus in a primate fibular osteotomy model^39^. Because bone formation is linked to resorption through coupling factors^40^,^41^, treatment with anti-resorption agents alone may results in simultaneous suppression of bone formation, which may compromise the efficacy of the drug^17^,^39^,^42^,^43^. Therefore, it will be more beneficial to identify an agent regulating both resorption and formation synchronously. We found 7E simultaneously downregulates osteoclast formation and upregulates osteoblast formation; therefore, it may have the effects of both denosumab (anti-RANKL mAb) and AMG 785 (anti-sclerostin mAb). We conclude that anti-IL-20 mAb is a potential therapeutic for healing bone fracture as well as protecting against osteoporotic bone loss.

## Methods

### Human amniotic fluid stem cell culture and osteoblast differentiation

Human amniotic fluid stem cells (hAFSCs)^29^,^30^,^31^ were a gift from Shiaw-Min Hwang, PhD (Bioresource Collection and Research Center, Hsinchu, Taiwan). The primary hAFSCs were cultured in α-MEM (Sigma-Aldrich) supplemented with 20% FBS (Hyclone) and 4 ng/ml of bFGF (Peprotech), and then incubated at 37 °C in 5% CO_2_. The hAFSCs at the 5th passage were grown to 70–90% confluence and shifted, for 14 and 28 days, to osteoblast differentiation medium (α-MEM supplemented with 10% FBS, 0.1 μM dexamethasone, 10 mM β-glycerol phosphate, 50 μM ascorbate) (Sigma-Aldrich) containing 200 ng/ml of IL-20 (R&D system), 2 μg/ml of 7E, or IL-20+7E. The culture medium was changed every 2 days for all differentiation experiments. Osteoblast differentiation was evaluated and confirmed using ALP staining (Sigma-Aldrich) 14 days and alizarin red S staining (Sigma-Aldrich) 28 days. ALP activity was measured using an ALP assay kit (ANASPCT) 14 days after the cells had been cultured.

### MC3T3-E1 cell culture and osteoblast differentiation

Mouse MC3T3-E1 preosteoblasts (American Type Culture Collection) were cultured in α-MEM and 10% FBS. Osteoblast differentiation from MC3T3-E1 cells was induced by culturing them in α-MEM supplemented with 10% FBS, 10 mM β-glycerol phosphate, and 50 μM of ascorbate containing 200 ng/ml of IL-20 (R&D system), 2 μg/ml of 7E, or IL-20+7E for 14 days. The osteoblast differentiation medium was replaced once every 2 days. The osteogenic activity was evaluated using ALP staining 14 days. ALP activity was measured using an ALP assay kit 14 days after the cells had been cultured.

### Reverse transcription polymerase chain reaction (RT-PCR)

Total RNA was extracted from cells using Trizol reagent (Invitrogen). The expression of IL-20, IL-20R1, IL-20R2, and IL-22R1 was analyzed using an amplified polymerase chain reaction (PCR) with gene-specific primers. β-actin was the internal control.

### Real-time quantitative (RTQ)-PCR

Total RNA was isolated. RT-PCR was done with reverse transcriptase (Clontech). OSX, RUNX2, Atf4, sclerostin, OPG, Wnt7a, Wnt7b, and Wnt3a expression was then amplified using SYBR Green with a real-time PCR system (LightCycler 480; Roche Diagnostics) with gene-specific primers (Supplementary Table S1). Quantitative analysis of mRNA was normalized with GAPDH as the housekeeping gene. Relative multiples of changes in mRNA expression were determined by calculating 2^−ΔΔC*t*^.

### Plasmids, transfection, and promoter activity assays

The construction of RUNX2 P1, RUNX P2 and OSX promoter were as previously described^28^,^44^,^45^,^46^,^47^. The promoter fragments were amplified by using PCR with specific primers and ligated into KpnI and SacI sites of pGL3-basic vector (Promega). HEK293 cells were plated in 6-well plates for luciferase assays and transfected. Each transfection assay was performed with 1 μg of plasmid DNA and 0.4 μg of the β-gal gene, which was used as an internal transfection efficiency control by using Lipofectamine 2000 (Invitrogen). Twenty- four hours after transfection, the medium was replaced with fresh medium and the cells were treated with IL-20 (200 ng/ml) for 24 h. Transfected cells were then collected for an analysis of luciferase activity assay system (Promega).

### Primary preosteoblast cell culture and osteoblast differentiation

Primary osteoblasts were isolated from the calvariae of 24-h-old mice using serial digestion as previously described. In brief, calvariae were dissected and subjected to sequential digestions in 2 mg/ml of collagenase A and 0.25% trypsin for 20, 40, and 90 min. Osteoblast differentiation from primary calvarial cells was induced by culturing them in α-MEM supplemented with 10% FBS, 0.1 μM of dexamethasone, 10 mM β-glycerol phosphate, and 50 μM of ascorbate for 21 days. The culture medium was replaced once every 2 days.

### Western blotting

MC3T3-E1 cells were stimulated with of mouse IgG (mIgG) (R&D system), 2 μg/ml of 7E, 200 ng/ml of IL-20, IL-20+mIgG, or IL-20+7E for 6 h. Western blotting was done with antibodies specific for β-catenin and anti-active β-catenin (Cell Signaling Technology). β-actin (GeneTex), used as an internal control, was detected using specific antibodies.

### Bone fracture model and treatments

All animal experiments were conducted according to the protocols based on the Taiwan National Institutes of Health (Taipei, Taiwan) standards and guidelines for the care and use of experimental animals. The research procedures were approved by the Animal Ethics Committee of National Cheng Kung University. The methods were carried out in accordance with the approved guidelines. All efforts were made to minimize animal suffering and to reduce the number of animals used. All animal experiments were as previously described^48^. Ten-week-old male BALB/c mice were given a surgical fracture. The experiments began 1 h after the fracture. The mice were divided into three groups (*n* = 8/group): bone fracture controls, bone fracture mice treated with 3 mg of mIgG/kg every 3 days, and bone fracture mice treated with 3 mg of 7E/kg every 3 days. To analyze the fracture healing of an IL-20R1 deficiency in the bone fracture model, both IL-20R1^+/+^ and IL-20R1^−/−^ mice underwent the fracture procedure (*n* = 8/group). All the mice were given an overdose of pentobarbital 21 or 42 days post-fracture. Serum was collected and serum IL-20 levels were determined using an IL-20 ELISA kit (Peprotech). The tibias were analyzed *in vivo* on a micro-CT (1076; SkyScan) with a high-resolution low-dose X-ray scanner. BMD at the fracture line, bone volume (BV/TV), trabecular bone thickness (Tb.Th), trabecular number (Tb.N.), and trabecular separation (Tb.Sp) were measured using bone histomorphometry (CT-analyzer software; SkyScan). Bone dynamic histomorphometric analyses for mineral apposition rate (MAR) and bone formation rate (BFR/BS) were performed and calculated following the protocol previously described^49^. Fracture healing was staged on the X-ray radiographs using a radiographic scoring system according to protocol previously described^50^. To calculate the number of osteoblasts and osteoclasts on the bone surface, the tibias were aseptically collected, cleaned of adherent soft tissue, frozen, and then sectioned for ALP and TRAP staining 21 days post-fracture.

### Patients

We recruited 165 patients (age range: 40–88 years) participating in a community-based chronic disease prevention study conducted from 2008 to 2010 by the Department of Family Medicine, National Cheng Kung University Hospital. Individuals who had a metabolic bone disease, were taking any medications likely to influence BMD, were bedridden, were alcohol dependent, were using steroids, or had a history of liver disease, stroke, hypertension, diabetes mellitus, arthrosclerosis, renal disease, or cancer were excluded from this study. BMD for all study participants was determined using dual energy X-ray absorptiometry (DXA) of the lumbar spine, hip, and femoral neck. We used World Health Organization criteria to categorize the participants into three groups based on DXA results: [i] normal BMD (T ≤ 1) (*n* = 29), [ii] patients with osteopenia (2.5 ≤ T ≤ 1) (*n* = 79), and [iii] patients with osteoporosis (T ≤ 2.5) (*n* = 57). We also collected, 5 days post-fracture, serum from patients with bone fracture (*n* = 10). Written informed consent was obtained. The Ethics Committee of National Cheng Kung University Hospital approved the study. The methods were carried out in accordance with the approved guidelines. Blood samples were collected. Serum levels of IL-20 and sclerostin were determined using an IL-20 ELISA kit and a sclerostin ELISA kit (R&D Systems).

### OVX-induced bone loss model and treatments

All animal experiments were conducted according to the protocols based on the Taiwan National Institutes of Health (Taipei, Taiwan) standards and guidelines for the care and use of experimental animals. The research procedures were approved by the Animal Ethics Committee of National Cheng Kung University. The methods were carried out in accordance with the approved guidelines. All efforts were made to minimize animal suffering and to reduce the number of animals used. All animal experiments were as previously described^9^. Fourteen-week-old female BALB/c mice were given an OVX or a Sham operation (Sham Control). The experiments began 7 days after surgery and the mice were divided into three groups (*n* = 5/group): Sham controls, OVX mice treated with 3 mg of mIgG/kg every 3 days, and OVX mice treated with 3 mg of 7E/kg every 3 days. All the mice were given an overdose of pentobarbital 8 weeks after the treatments had begun. In another experiment, to analyze the protective effect of an IL-20R1 deficiency in an OVX-induced bone loss model, IL-20R1^+/+^ and IL-20R1^−/−^ mice were OVX or Sham-operated on (*n* = 5/group). All the mice were given an overdose of pentobarbital 8 weeks after the surgery. Serum was collected from blood that had been centrifuged at 2000 rpm for 10 min at 4 °C. Serum levels of IL-20 and sclerostin were determined using an IL-20 ELISA kit and a sclerostin ELISA kit. To calculate the number of osteoblasts and osteoclasts on the bone surface, the tibias were aseptically collected, cleaned of adherent soft tissue, frozen, and then sectioned for ALP and TRAP staining 8 weeks after treatment.

### Statistical analysis

The correlation between IL-20 and sclerostin was analyzed using SPSS 15.0 for Windows. Prism 5.0 (GraphPad Software, San Diego, CA) was also used for the statistical analysis. A one-way analysis of variance (ANOVA) nonparametric Kruskal-Wallis test was used to compare the data between groups. Post hoc comparisons were done using Dunn’s multiple comparison test. Data are means ± standard deviation (SD). Significance was set at P < 0.05.

## Additional Information

**How to cite this article**: Hsu, Y.-H. *et al.* Anti-IL-20 monoclonal antibody promotes bone fracture healing through regulating IL-20-mediated osteoblastogenesis. *Sci. Rep.* **6**, 24339; doi: 10.1038/srep24339 (2016).

## Supplementary Material

Supplementary Information
