# Supplementary material for: Anti-IL-20 monoclonal antibody promotes bone fracture healing through regulating IL-20-mediated osteoblastogenesis
Source: Sci Rep. 2016 Apr 14;6:24339. doi: 10.1038/srep24339 (PMC4830982; doi:10.1038/srep24339)
Supplement: Supplementary Information [file srep24339-s1.pdf]

Anti-IL-20 monoclonal antibody promotes bone fracture healing through regulating IL-20-mediated osteoblastogenesis

Yu-Hsiang Hsu, Yi-Shu Chiu, Wei-Yu Chen, Kuo-Yuan Huang, I-Ming Jou, Po-Tin Wu, Chih-Hsing Wu, & Ming-Shi Chang

Supplementary Information

Supplementary Table S1. Sequences of forward and reverse primers used in PCR analysis

**hRunx2**	Forward	CGCATTCCTCATCCCAGTAT
	Reverse	GACTGGCGGGGTGTAAGTAA
**hOsx**	Forward	CCCTGCTTGAGGAGGAAGTT
	Reverse	CACTGGGCAGACAGTCAGAA
**hATF4**	Forward	AAACCTCATGGGTTCTCCAG
	Reverse	GGGCTCATACAGATGCCACT
**hSclerostin**	Forward	ACCACCCCTTTGAGACCAAAG
	Reverse	GGTCACGTAGCGGGTGAAGT
**hβ-actin**	Forward	GCTGGAAGGTGGACAGCGAG
	Reverse	TGGCATCGTGATGGACTCCG
**mOPG**	Forward	TGTCCAGATGGGTTCTTCTCA
	Reverse	CGTTGTCATGTGTTGCATTTCC
**mSclerostin**	Forward	AAGCCGGTCACCGAGTTGGT
	Reverse	GTGAGGCGCTTGCACTTGCA
**mOsx**	Forward	TGAGGAAGAAGCCCATTCAC
	Reverse	GGTAAAGCGCTTGGAACAGA
**mAtf4**	Forward	CGGCACACGCGGTTTTACAA
	Reverse	CAGTAGCGCTTTAGACACTC
**mWnt3a**	Forward	CATGCACCTCAAGTGCAAATG
	Reverse	TGAGGAAATCCCCGATGGT
**mWnt7a**	Forward	TGGATGCCCGGGAGATC
	Reverse	CCGACCCGCCTCGTTATT
**mWnt7b**	Forward	TTCTGGAGGACCGCATGAA
	Reverse	GGTCCAGCAAGTTTTGGTGGTA
**mβ-actin**	Forward	CCTCTATGCCAACACAGTGC
	Reverse	CACACAGAGTACTTGCGCTC

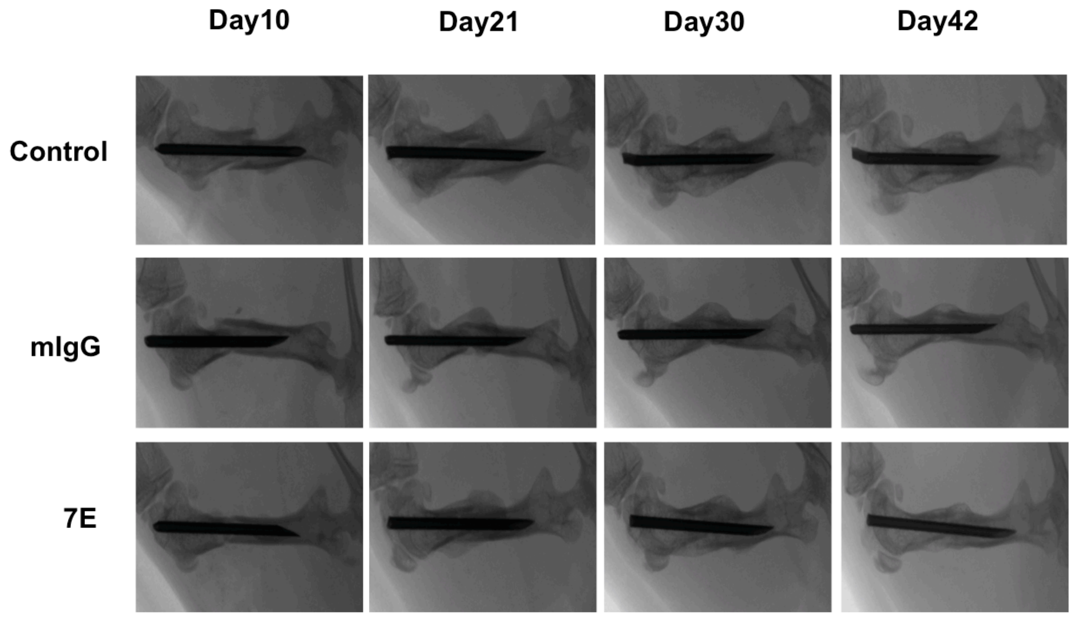

Supplementary Figure S1. Representative radiographs of right femurs. Radiographs of fractured femurs from bone fracture control mice, followed by 3 mg mIgG/kg/3 d-treated mice, and 3 mg 7E/kg/3 d-treated mice (*n* = 4/group). The radiographic time course is shown at the fracture line for the mice in each group. Data are representative of three independent experiments.

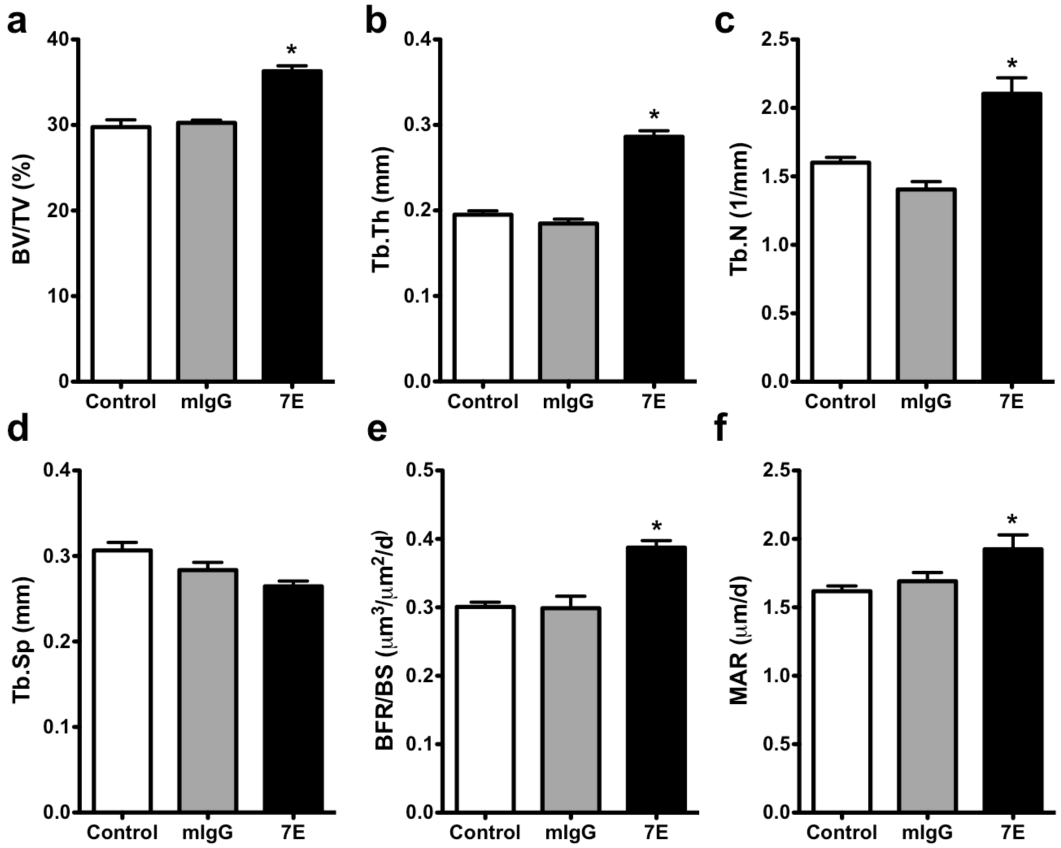

Supplementary Figure S2. 7E promoted fracture healing by increasing bone formation in the mouse fracture model. Micro-CT-based measurements of fracture calluses were performed at 21 days post-fracture on the control (no treatment), 3 mg mIgG/kg/3 d treatment, and 3 mg 7E/kg/3 d treatment (n = 8/group). (a) The bone volume relative to total tissue volume (BV/TV) ratio indicates the fraction of mineralized tissue in the total callus volume. (b) Tb.Th indicates the trabecular bone thickness. (c) Tb.N indicates the trabecular bone number in the total callus tissue. (d) Tb.Sp indicates the dimension of trabecular bone separation. (e-f) Analysis of dynamic bone histomorphometric parameters (MAR and BFR/BS) in the distal femur collected from the groups of mice indicated. Values are means ± SEM. Data are representative of three independent experiments. *$P < 0.05$ versus mIgG controls.

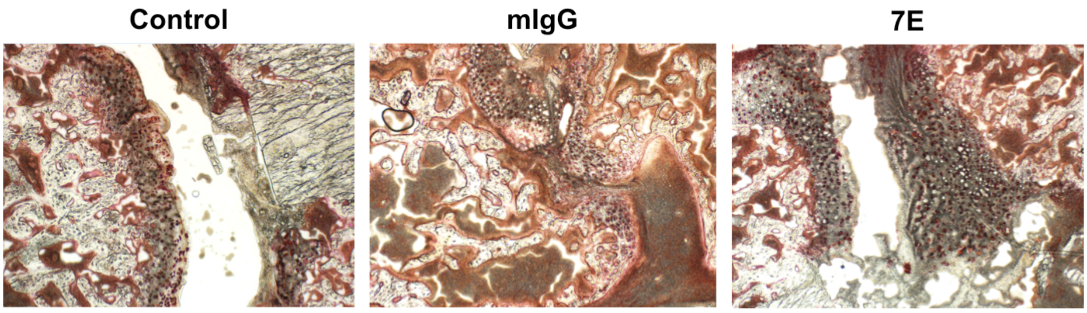

Supplementary Figure S3. ALP staining of osteoblasts in fracture callus. Representative figures of ALP staining of osteoblasts in the fracture callus of mice 21 days post-fracture from the untreated control, 3 mg mIgG/kg/3 d treatment, and 3 mg 7E/kg/3 d treatment (n = 8/group). Images of these individual slices were taken from the central region of each callus. Data are representative of three independent experiments.

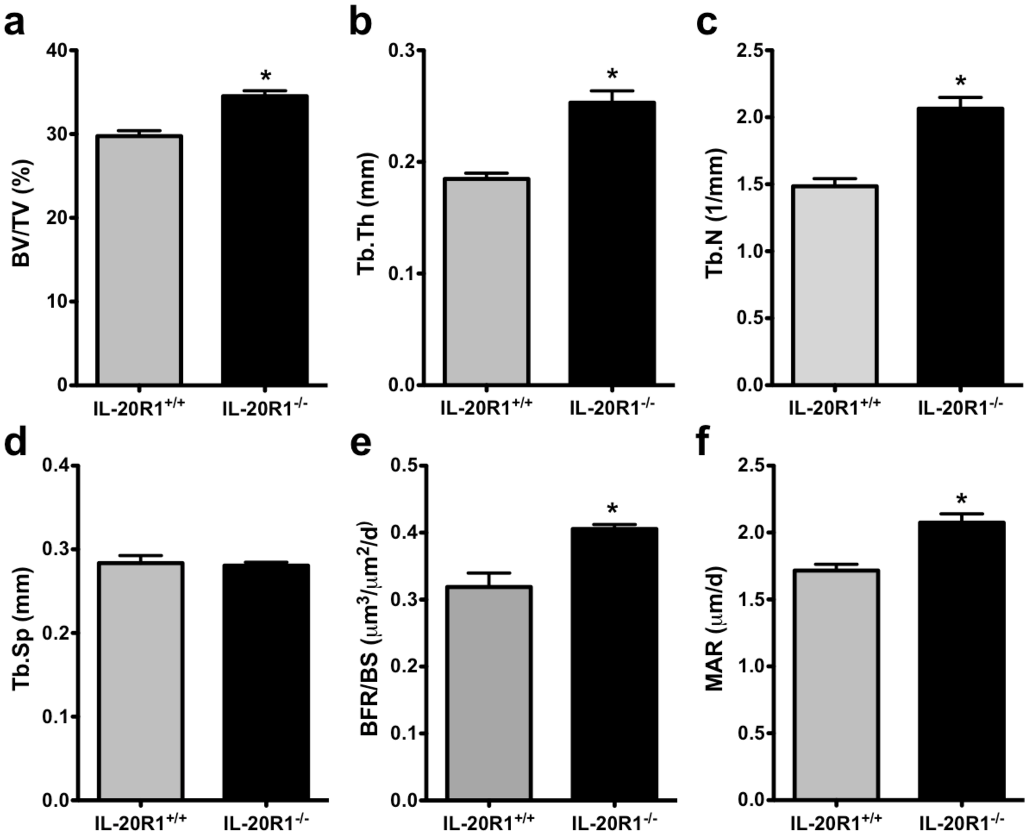

Supplementary Figure S4. IL-20R1 deficiency increased osteoblast differentiation and promoted fracture healing by increasing BMD in the mouse fracture model. Micro-CT-based measurements of fracture calluses at 21 days post-fracture from IL-20R1$^{+/+}$ and IL-20R1$^{-/-}$ mice (n = 8/group). (a) The bone volume relative to total tissue volume (BV/TV) ratio indicates the fraction of mineralized tissue in the total callus volume. (b) Tb.Th indicates the trabecular bone thickness. (c) Tb.N indicates the trabecular bone number in the total callus tissue. (d) Tb.Sp indicates the dimension of trabecular bone separation. (e-f) Analysis of dynamic bone histomorphometric parameters (MAR and BFR/BS) in the distal femur collected from the groups of mice indicated. Values are means ± SEM. Data are representative of three independent experiments. *$P < 0.05$ versus IL-20R1$^{+/+}$ mice.

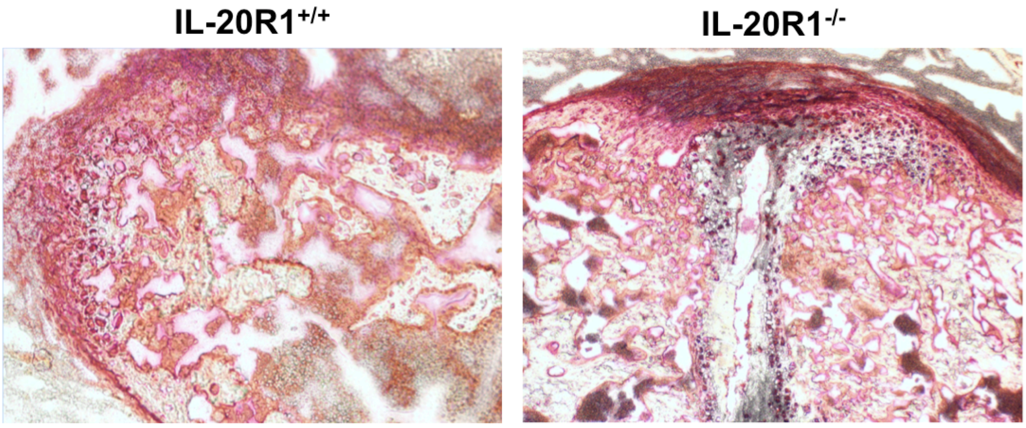

Supplementary Figure S5. ALP staining of osteoblasts in fracture callus. Representative figures of ALP staining of osteoblasts in the fracture callus at 21 days post-fracture from IL-20R1$^{+/+}$ and IL-20R1$^{-/-}$ mice (n = 8/group). Images of these individual slices were taken from the central region of each callus. Data are representative of three independent experiments.

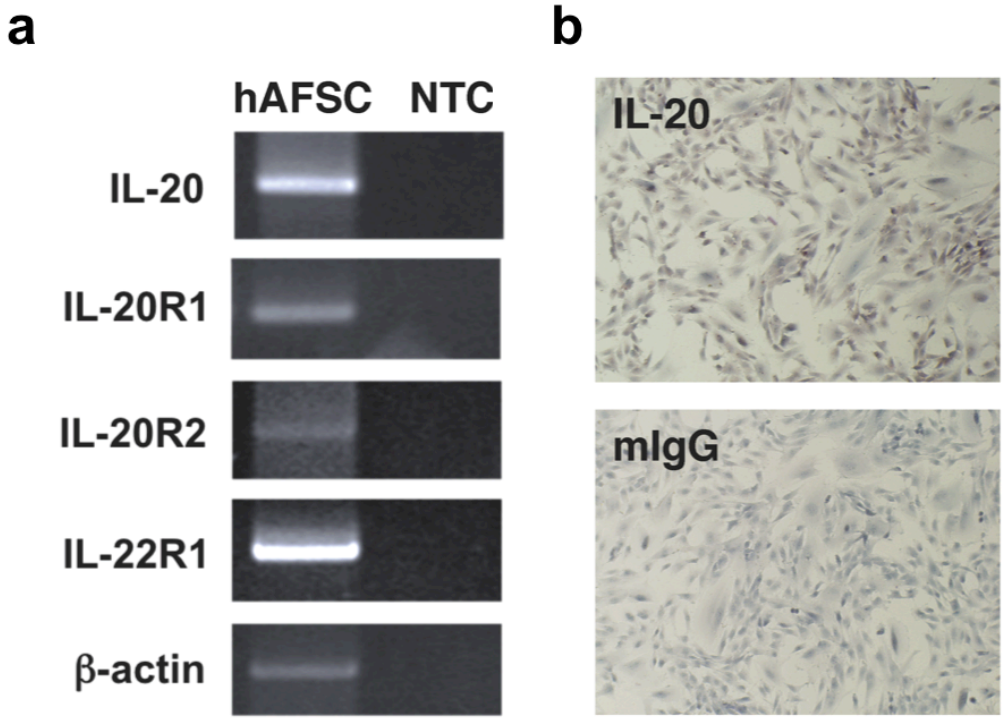

Supplementary Figure S6. Expression of IL-20 and its receptors in hAFSCs. (a) The expression of IL-20 and its receptors in hAFSCs was analyzed using RT-PCR with specific primers. NTC: non-template control. (b) hAFSCs were stained for the expression of IL-20 and mIgG1. Data are representative of three independent experiments.
